# Rapid and accurate method for quantifying busulfan in plasma samples by isocratic liquid chromatography-tandem mass spectrometry (LC-MS/MS)

**DOI:** 10.1515/almed-2022-0016

**Published:** 2022-06-13

**Authors:** Yolanda Villena-Ortiz, Laura Castellote-Bellés, Luisa Martinez-Sanchez, María I. Benítez-Carabante, Marta Miarons, Jaume Vima-Bofarull, Raquel Barquin-DelPino, Rosanna Paciucci, Francisco Rodríguez-Frías, Roser Ferrer-Costa, Ernesto Casis, Joan López-Hellín

**Affiliations:** Biochemistry Department, Vall d’Hebron Institut de Recerca (VHIR), Vall d’Hebron University Hospital, Barcelona, Spain; Departament de Bioquimica i Biologia Molecular, Universitat Autonoma de Barcelona, Bellaterra, Spain; Department of Pediatric Hematology and Oncology, Vall d’Hebron Institut de Recerca (VHIR), Vall d’Hebron University Hospital, Barcelona, Spain; Pharmacy Department, Vall d’Hebron Institut de Recerca (VHIR), Vall d’Hebron University Hospital, Barcelona, Spain

**Keywords:** hematopoietic stem cell transplantation, mass spectrometry platform, method validation, therapeutic drug monitoring

## Abstract

**Objectives:**

Administration of busulfan is extending rapidly as a part of a conditioning regimen in patients undergoing hematopoietic stem cell transplantation (HSCT). Monitoring blood plasma levels of busulfan is recommended for identifying the optimal dose in patients and for minimizing toxicity. The aim of this research was to validate a simple, rapid, and cost-effective analytical tool for measuring busulfan in human plasma that would be suitable for routine clinical use. This novel tool was based on liquid chromatography coupled to mass spectrometry.

**Methods:**

Human plasma samples were prepared using a one-step protein precipitation protocol. These samples were then resolved by isocratic elution in a C18 column. The mobile phase consisted 2 mM ammonium acetate and 0.1% formic acid dissolved in a 30:70 ratio of methanol/water. Busulfan-d_8_ was used as the internal standard.

**Results:**

The run time was optimized at 1.6 min. Standard curves were linear from 0.03 to 5 mg/L. The coefficient of variation (%CV) was less than 8%. The accuracy of this method had an acceptable bias that fell within 85–115% range. No interference between busulfan and the interfering compound hemoglobin, lipemia, or bilirubin not even at the highest concentrations of compound was tested. Neither carryover nor matrix effects were observed using this method. The area under the plasma drug concentration-time curves obtained for 15 pediatric patients who received busulfan therapy prior to HSCT were analyzed and correlated properly with the administered doses.

**Conclusions:**

This method was successfully validated and was found to be robust enough for therapeutic drug monitoring in a clinical setting.

## Introduction

Busulfan (1,4-butanediol dimethanesulphonate) is an antineoplastic, bifunctional, and alkylating agent. This reagent induces the formation of covalent DNA adducts and DNA breaks and is known to inhibit replication [1]. Busulfan is often administered intravenously to patients with acute myeloid leukemia and thalassemia as a myeloablative agent prior to hematopoietic stem cell transplantation (HSCT) [2], [3], [4]. As with other chemotherapeutic agents, busulfan has a narrow therapeutic index and a marked interindividual variability in pharmacokinetic parameters [5, 6]. The area under the plasma drug concentration-time curve (AUC) is used to prospectively determine the maximum tolerated systemic exposure to busulfan [7]. High AUC is associated with an increased risk of adverse events such as veno occlusive disease [8], [9], [10]. Therefore, precise monitoring of the plasma concentrations of busulfan is recommended to determine the correct dose for a patient in order to improve efficacy, reduce toxicities, and optimize the therapy [11].

Several analytical methods have been employed to measure busulfan in plasma. These methods make use of tools such as gas chromatography coupled with mass spectrometry [12, 13] and high-pressure liquid chromatography coupled with ultraviolet spectroscopy [14] or with a fluorescence detector [15]. More recently, liquid chromatography coupled with tandem mass spectrometry (LC-MS/MS) methodologies have been developed [16], [17], [18], [19], [20], [21], [22], [23], [24], [25], [26], [27] (Table 1). LC-MS/MS, in particular, combines high analytical specificity and sensitivity with a lower cost, significantly shorter turnaround times, and a simplified workflow [28]. Nevertheless, current techniques for determining the blood plasma levels of busulfan are time consuming and lacked a well-detailed evaluation of interfering compounds and a short stability study. The aim of this research was to validate a simple, rapid, and cost-effective analytical tool for measuring busulfan in human plasma that would be suitable for routine clinical use.

**Table 1:** Differences between our developed LC-MS/MS method and published LC-MS/MS methods over last 10 years for quantifying busulfan.

Reference (order#)	Run time, min	Elution type	Mobile phase	Column	Linearity, mg/L	Internal standard	Sample preparation	Injection volume, μL	Total time of analysis^a^
Deng et al., 2016 [17]	8	Gradient	A: 20 mM ammonium acetate and 0.5% formic acid in water; B: 20 mM ammonium acetate and 0.5% formic acid in methanol	Supelcosil LC-18 column (50 × 4.6 mm, 5 μm)	0.05–2.5	Busulfan-d_8_	Protein precipitation	20	140
French et al., 2014 [25]	4	Gradient	A: 2 mM ammonium acetate and 0.1% formic acid in water; B: 2 mM ammonium acetate and 0.1% formic acid in methanol	Kinetex C18 column (100 × 3 mm, 2.6 mm)	0.01–2	Busulfan-d_8_	Protein precipitation and dilution	10	95
Jinjie et al., 2020 [18]	5	Gradient	A: 10 mM ammonium acetate and 0.1% formic acid in acetonitrile; B: acetonitrile	Hypersil Gold C18 column (150 × 2.1 mm, 5 μm)	0.01–10	Busulfan-d_8_	Protein precipitation	5	95
Matar et al., 2020 [19]	2	Isocratic	Methanol/20 mM ammonium acetate buffer (90:10, v/v)	Acquity UPLC BEH C18 column (50 × 2.1 mm, 1.7 μm)	0.025–2	Busulfan-d_8_	Protein precipitation and sample evaporation and reconstitution	10	80
Moon et al., 2014 [20]	10	Gradient	A: 2 mM ammonium acetate and 0.1% formic acid in water; B: 2 mM ammonium acetate and 0.1% formic acid in methanol	XBridge™ C18 column (100 × 2.1 mm, 3.5 μm)	0.025–5	Glipizide	Protein precipitation	2	170
Nadella et al., 2016 [21]	2	Isocratic	Acetonitrile/10 mM ammonium formate buffer (80:20, v/v)	Kinetex C18 column (50 × 2.1 mm, 2.6 μm)	0.04–2	Busulfan-d_8_	Protein precipitation	10	50
Punt et al., 2017 [26]	4.5	Gradient	A: 0.1% formic acid in water; B: 0.1% ammonium acetate in acetonitrile	Acquity UPLC BEH C18 column (50 × 2.1 mm, 1.7 μm)	0.01–10	Busulfan-d_8_	Protein precipitation and dilution	3	105
Schofield et al., 2019 [22]	8	Gradient	A: 10 mM ammonium formate in water; B: methanol; C: acetonitrile/2-propanol/acetone (6:3:1, v/v)	Hypersil Gold C18 HPLC column (50 × 3 mm, 5 μm)	0.01–5	Busulfan-d_8_	Protein precipitation	25	140
Xiao et al., 2018 [24]	3	Gradient	A: 10 mM ammonium acetate and 0.1% formic acid in water; B: 0.1% formic acid in acetonitrile	Luna C8 (2) HPLC column (50 × 2 mm, 3 μm)	0.01–5	Busulfan-d_8_	Protein precipitation and dilution	3	80
Current	1.6	Isocratic	2 mM ammonium acetate and 0.1% formic acid in methanol/water (30:70, v/v)	Mediterranean Sea 18 UPLC Column (50 × 2.1 mm, 1.8 µm)	0.03–5	Busulfan-d_8_	Protein precipitation	2	45

^a^Total time of analysis includes extraction methodology and analytical run time. This time is approximate and has been calculated based on the pretreatment described and including standards, controls, and five samples.

## Materials and methods

### Reagents

Busulfan, busulfan-d_8_, analytical grade ammonium acetate, and bilirubin were purchased from Sigma-Aldrich (St. Louis, MO, USA). Busulfan-d_8_ was supplied at a concentration of 100 μg/mL in methanol. Ultra LC/MS grade acetonitrile (ACN) was purchased from Honeywell (Charlotte, NC, USA). Ultra LC/MS grade methanol was purchased from Fisher Scientific (Waltham, Massachusetts, USA). Analytical grade formic acid was purchased from PanReac AppliChem (Barcelona, Spain). Intralipid^®^ was provided by Fresenius Kabi España (Barcelona, Spain). Plasma and hemolyzed plasma were collected from patients not treated with busulfan.

### Equipment and conditions

The plasma samples were analyzed using a Nexera X2 liquid chromatograph coupled to a Shimadzu LCMS-8050 tandem mass spectrometer (Shimadzu, Kyoto, Japan) equipped with an electrospray ionization source. The chromatographic analyses were performed with 50 × 2.1 mm, 1.8 µm, Mediterranean Sea 18 UPLC Column from Teknokroma^®^. The column was set to a temperature of 40 °C. The analytes were eluted at a flow rate of 0.3 mL/min using an isocratic elution step that was 1.6 min long. The mobile phase consisted 2 mM ammonium acetate and 0.1% formic acid in methanol/water (30:70, v/v).

The mass detector was set to a positive ion mode. Selected reaction monitoring (SRM) was carried out using scan width of 0.5 *m*/*z*. The mass parameters for the analyte and the internal standard are shown in Table 2. The interface temperature was set to 300 °C, the desolvation line temperature was set to 250 °C, and the heat block temperature was set to 350 °C. Nitrogen gas was used as nebulizing gas at a flow rate of 3 L/min. Nitrogen was also used as the drying gas at a flow rate of 10 L/min. Air was used as the heating gas at a flow rate of 10 L/min. The data were analyzed using LabSolutions software (Shimadzu).

**Table 2:** Mass spectrometer detector settings for analyte and internal standard SRM transitions.

	Parent ion (*m*/*z*) and linear formula	Product ion (*m*/*z*) and linear formula	Q1 and Q3 voltages, V	Collision energy, V	Retention time, min
Analyte	264.1 [CH_3_SO_2_O(CH_2_)_4_OSO_2_CH_3_-NH_4_]^+^	151.0 [CH_3_SO_2_O(CH_2_)_4_-H]^+^	12/16	11	0.78
Internal standard	272.1 [CH_3_SO_2_O(CD_2_)_4_OSO_2_CH_3_-NH_4_]^+^	159.0 [CH_3_SO_2_O(CD_2_)_4_-H]^+^	13/17	13	0.77

### Sample preparation

#### Stock solutions, calibration standards, and quality control samples

Separate stock solutions of 500 mg/L of busulfan and 500 mg/L of internal standard were both prepared in ACN and stored at −20 °C. A blank, a zero, and six nonzero calibration standards with concentrations 5, 1, 0.5, 0.25, 0.08, and 0.03 mg/L of busulfan were prepared in plasma. Five quality control (QC) samples containing the following concentration of busulfan were prepared in plasma: 4.0 mg/L (HIGH), 1.5 mg/L (MEDIUM), 0.75 mg/L (LOW), 0.09 mg/L (VERY LOW) and 0.03 mg/L (low limit of quantification, LLOQ). The total amount of stock solutions added to the plasma was less than 5% of the final volume.

#### Sample pretreatment

Plasma samples were prepared by protein precipitation with ACN. A 100 μL aliquot of plasma, standard, or QC sample was mixed with 100 μL of internal standard working solution (containing 0.1 mg/L of busulfan-d_8_). The samples were adjusted by adding 600 µL of ACN. The samples were then placed in a vortex mixer for 1 min and centrifuged at 10,000×*g* at room temperature for 6 min. Two microliters of the resulting supernatant were injected into the LC-MS/MS system.

### Linearity

To determine the linearity of the method, the calibration curves were analyzed once a day for 5 days. The calibration curves were obtained by dividing the peak area of the analyte by the peak area of the internal standard and plotting this value against the nominal concentration of the sample. The bias was allowed to be within 15% for all calibration standards (within 20% at the LLOQ). The linearity of the calibration standards was considered confirmed when the 1/x^2^ weighted linear regression curve fitted to the data resulted in a correlation coefficient of more than 0.995 [29].

### Analytical sensitivity

An LLOQ evaluation was conducted as a part of the precision and accuracy assessment of the calibration range. The LLOQ for busulfan quantification was determined as the minimal concentration of busulfan in plasma that could be quantified with a ±20% deviation between measured and nominal concentration, in accordance with FDA and EMA recommendations [30, 31].

### Accuracy and precision

The validation assays for accuracy and precision were performed once a day for 3 days. Five replicates of each QC sample (LLOQ, VERY LOW, LOW, MEDIUM, and HIGH) were assessed [31]. The bias and %CV were calculated from the data from each run and for each concentration. Uncertainty of measurement (UoM) was calculated as 1.96 × %CV. The acceptance criteria of data included a trueness within ±15% bias of the nominal values and a precision within ±15% CV [30, 31].

To assess the trueness of busulfan measurement, eight samples were analyzed in duplicate from the Stichting Kwaliteitsbewaking Medische Laboratoriumdiagnostiek (SKML) External Quality Assurance Scheme (range of 0.71–3.47 mg/L). The performance was deemed acceptable if results were within ±15% of nominal concentrations.

### Selectivity and carryover

The selectivity of our method was evaluated by comparing the responses of the LLOQ samples with the responses of the blanks. The blanks were prepared from 50 busulfan-free plasma samples. The confounding effects of hemoglobin, lipemia, and bilirubin were assessed by analyzing samples with a fixed amount of busulfan (1.5 mg/L) in the presence of increasing concentrations of hemoglobin, Intralipid^®^, or bilirubin. The percentage change was calculated as: %Interference=100 × (C_I_−C_0_)/C_0_, where C_0_ was the busulfan containing sample in the absence of the confounding factor and C_i_ was the busulfan containing sample in the presence of the interfering factor. A significant interference was defined as a difference in %Interference measurement that exceeded the total error of busulfan at 1.5 mg/L (calculated as |bias%| + 1.96 × CV%).

The carryover was assessed by comparing the peak area of a blank sample injected immediately after a 4 mg/L sample and the peak area of a blank sample injected immediately after the 5 mg/L standard with the LLOQ peak area. The measurements were performed in duplicate for 5 days. A difference in value of less than 5% was considered acceptable [29].

### Matrix effect, recovery, and process efficiency

The matrix effect (ME), analyte recovery (RE), and process efficiency (PE) ratios were measured at two concentrations of busulfan (0.09 mg/L and 1.5 mg/L) based on the procedure described by Matuszewski et al. [32]. The ME was also calculated at 4 mg/L. Three sets of samples were prepared to assess ME, RE, and PE as follows: a) set 1 was neat samples in ACN solvent; b) set 2 was spiked into blank extracted samples; and c) set 3 was regular extracted plasma samples. ME was determined by comparing set 2 to set 1, RE by comparing set 3 to set 2, and PE by comparing set 3 to set 1. Six independent plasma pools were analyzed in triplicate to determine the ME, RE, and PE.

The ME and the PE were considered acceptable if values within ±15% were observed. The RE is acceptable when data are consistent, precise, and reproducible.

### Stability assessments

LOW and HIGH QC samples were freshly prepared and divided into 0.1 mL aliquots. All analyses were performed in triplicate. To determine the stability of busulfan at room temperature, the QC samples were analyzed after storage for 24, 48, and 72 h at room temperature [31]. To analyze the sample stability during refrigerator storage, the QC samples were analyzed after 24 h, 72 h, and 7 days at 4–8 °C. The stability of samples stored at −20 °C was also analyzed at 30 and 60 days. To determine the stability during freezing and thawing, individual QC samples were thawed and frozen again at −20 °C once a day for 3 days and analyzed after each thawing step. To determine the sample stability in the autosampler, the supernatants of QC samples were kept in the autosampler for 24 h and then analyzed.

In light of previously reported instability of busulfan, short stability study was performed. To analyze short stability, QC samples were analyzed after 2, 4, 6 and 8 h at room temperature and at 4–8 °C.

### Clinical application

All patients included in the study were pediatric patients who were being administered busulfan intravenously once a day for 4 days over a 3 h period, together with either fludarabine [33], fludarabine and thiotepa, cyclophosphamide [34] or cyclophosphamide and melphalan [35]. The initial dose administered was based on the AUC target, subsequently adjusted for each patient’s body weight and following individualized dosing nomograms, as described previously [36]. The dosing nomograms were designed to achieve either a myeloablative or a nonmyeloablative conditioning regimen. All patients who were undergoing HSCT for nonmalignant diseases and patients with malignant diseases when the donor was unrelated received *ex vivo* T-cell depletion with either thymoglobulin or alemtuzumab. Neither paracetamol nor azoles were administered during the busulfan treatment. Levetiracetam was administered as a prophylactic anticonvulsant therapy.

To measure the busulfan concentration in plasma of children who had received therapy prior to undergoing HSCT, blood samples (n=75) were collected in K3-EDTA Vacutainer tubes (Becton Dickinson, Milan, Italy). The samples were then transported at room temperature, centrifuged at 2,500×*g* for 5 min at room temperature, and frozen within 1 h of arriving to the laboratory.

In order to estimate the AUC, blood samples were collected for analysis immediately prior to chemotherapy infusion (time 0), at the point when the infusion ended, and 1 h, 2 h, and 3 h after the infusion step [36]. About 3–5 mg/kg/24 h of busulfan were the initial dosage chosen, regardless of age (3 mg/kg/24 h in three patients; 4 mg/kg/24 h in four patients; and 5 mg/kg/24 h in eight patients). Starting from day 2, the busulfan dosage was adjusted based on the results of the previous day’s therapeutic drug monitoring.

The protocol for sample collection, storage, and analysis was approved by the Vall d’Hebron Hospital Institutional Review Board (EOM(AG)027/2021(5825)). The study was conducted in accordance with the Spanish and European law, and the principles of the Declaration of Helsinki.

## Results

The mean retention times were 0.78 min for busulfan and 0.77 min for busulfan-d_8_. Acceptable peak shapes that had neither tailing nor interference were observed (Supplemental Figure 1).

### Linearity

All calibration curves from the validation step were linear over a range of 0.03–5 mg/L (mean of 0.9958) (Supplemental Figure 2). None of the standards were discarded during the validation step.

### Analytical sensitivity

This LC-MS/MS method could be used to precisely quantify busulfan at a concentration of 0.03 mg/L (n=40). The signal-to-noise ratio observed at 0.03 mg/L busulfan was five times higher than the ratio observed with the blank alone.

### Accuracy and precision

Both intraday and interday precision for all five QC samples were <7.2% (Supplemental Table 1). The mean bias for all concentrations was under 10% with respect to the nominal concentration (n=15 for each QC sample). The UoM was calculated to be between 7.25 and 12.6%.

The results obtained on SKML External Quality Assurance Scheme demonstrated a good agreement with the assigned values (n=16; y=0.99x − 0.05 mg/L; r^2^>0.97). All individual results obtained were within the permitted deviation of ±15% from the LC-MS/MS consensus values. The observed mean bias was +4.1%.

### Selectivity and carryover

**Figure 1:**
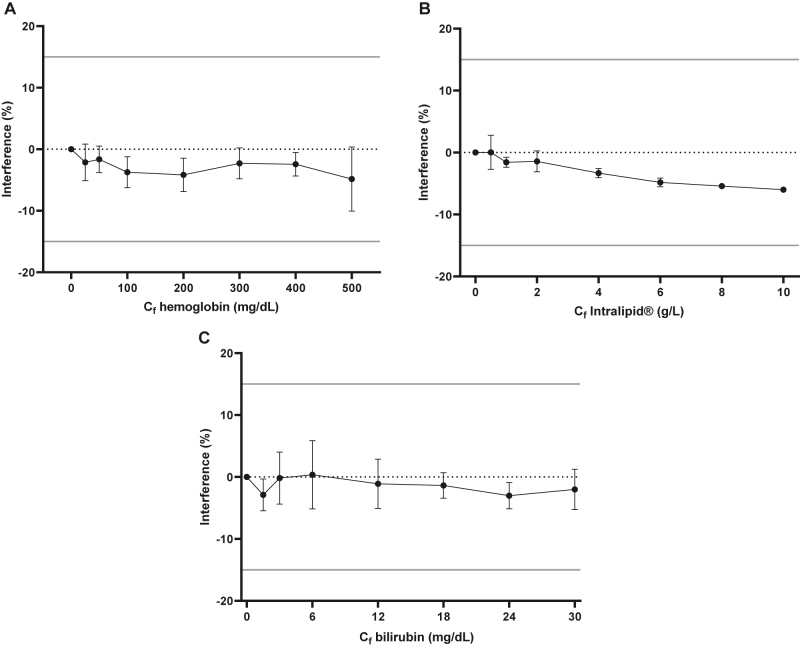
Interferographs of hemoglobin (A), lipemia (B), and bilirubin (C) at a busulfan concentration of 1.5 mg/L (mean and standard deviation). C_f_ is the concentration in solutions of the interferent: hemoglobin, Intralipid^®^, or bilirubin.

No interfering components were revealed in the relevant mass transitions from blank patient samples (n=50). The method was validated with plasma samples containing hemoglobin, high concentration of Intralipid^®^, or high levels of bilirubin (Figure 1). The mean bias of %Interference measurement was −3.1%, −3.2%, and −1.5%, respectively. The maximum bias for all concentrations of interferent was under a total error of busulfan at 1.5 mg/L, 12.8%.

No carryover was observed for either busulfan (n=10) or internal standard (n=10).

### ME, RE, and PE

All three tests were consistent over the tested concentration ranges (Table 3). The mean internal standard normalized matrix effect (ME IS-normalized) for busulfan concentrations of 0.09 mg/L, 1.5 mg/L, and 4 mg/L were calculated to be 92.9%, 100.2%, and 99.8%, respectively. The RE ratios were 94.6% and 103.3% for 0.09 mg/L and 1.5 mg/L samples, and the PE ratios were 98.2% and 97.1%, respectively.

**Table 3:** Results of the matrix assessment. All results are expressed as normalized percent matrix bias.

Busulfan concentration, mg/L	Matrix effect, %				Recovery, %				Process efficiency, %			
	n	Mean, %	SD, %	CV, %	n	Mean, %	SD, %	CV, %	n	Mean, %	SD, %	CV, %
0.09	18	92.9	3.5	3.7	18	94.6	1.7	1.8	18	98.2	3.7	3.7
1.5	18	100.2	4.3	4.3	18	103.3	4.9	4.8	18	97.1	2.6	2.7
4	18	99.8	10.1	10.1	n.d.				n.d.			

n, sample size; SD, standard deviation; CV, coefficient of variation; n.d., not determined.

### Stability

**Figure 2:**
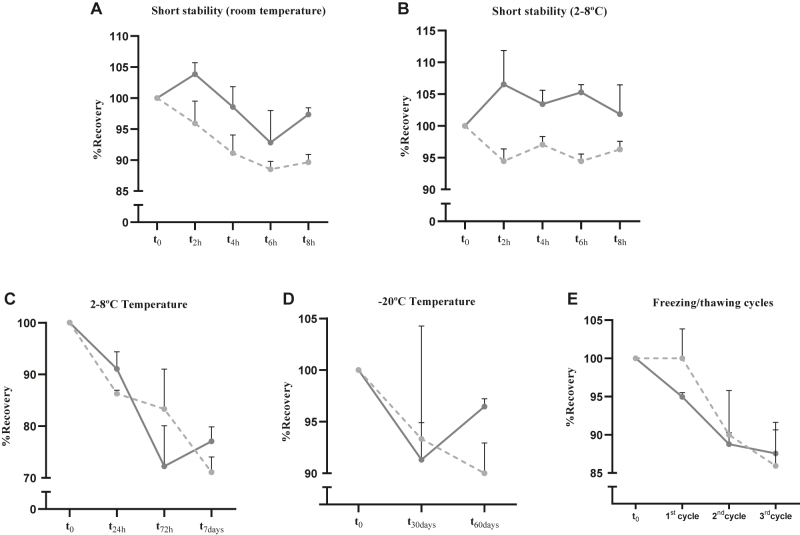
Stability of plasma busulfan on storing at room temperature (A), 2–8 °C (B, C), and −20 °C (D); and subjected to freeze-thaw conditions (E). Mean and standard deviation are shown by condition. Continuous line represents the HIGH QC sample; dotted lines represent the LOW QC sample.

Busulfan is stable in plasma for at least 8 h at room temperature, for at least 24 h at 2–8 °C and up to 2 months at −20 °C (Figure 2). The stability tests showed that busulfan was not stable in plasma at room temperature for 24, 48, or 72 h (data not shown). The busulfan concentration decreased by less than 15% after 3 freezing/thawing cycles (data not shown). The supernatants of busulfan samples in plasma were stable in the autosampler for 24 h: only slight decreases of 13.7 and 8.9% of busulfan were observed for 0.09 and 1.5 mg/L samples, respectively. Meanwhile, a more substantial decrease was observed at 7 days: 18.9% for 0.09 mg/L samples and 16.8% for 1.5 mg/L samples.

### Clinical application

**Figure 3:**
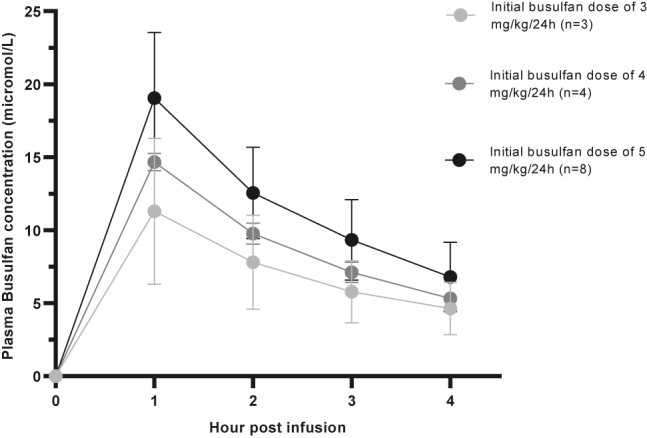
Distribution and change of plasma busulfan concentrations in clinical samples after initial busulfan doses of 3 (n=3), 4 (n=4), and 5 (n=8) mg/kg/24 h. The middle point represents the mean value, and the Whiskers represents the standard deviation. 1 mg/L=4.06 μmol/L.

Finally, we monitored the plasma busulfan concentrations in 15 pediatric patients (n=75 samples; seven females; mean age: 4.4 ± 3.5 years, range 6 months–12 years). The sample replicates verified the reliability of the reported results (data not shown). The AUC results were consistent with respect to the administered dose (Figure 3). After initial busulfan doses of 3 (n=3 patients), 4 (n=4 patients), and 5 (n=8 patients) mg/kg/24 h, the AUCs ranged from 931.8 to 1780.9, from 1653.9 to 1795.4, and from 1531.9 to 3230.0 µM/min, respectively.

## Discussion

Here, we present a rapid, simple, and robust LC-MS/MS method for busulfan monitoring in plasma samples on a mass spectrometer that would be available to the majority of clinical laboratories. The method described here meets the established criteria for accuracy and precision and is validated for clinical applications.

Minimizing run times is important during therapeutic drug monitoring. This is because test results need to be reported promptly in order to facilitate timely decision-making regarding changes in treatment. The method described here has several advantages over existing methods for monitoring busulfan by LC-MS/MS [16], [17], [18], [19], [20], [21], [22], [23], [24], [25], [26], [27], which include lower run times compared to previously described methods (Table 1). The results can be reported within just 1 h and 30 min. This figure includes sample transport and reception times, analyzer and sample preparation times, and the time dedicated to verify results. Regarding the sample preparation procedures cited in Table 1, we reduced the analytical time to 45 min. Another important difference is that this method employs an isocratic elution protocol with a single mobile phase. As a consequence, this protocol requires less reagent consumption and makes the method simpler to use. In comparison with other isocratic elution methods for quantifying busulfan [19, 21], our analytical measurement range is wider and diminishes the number of samples to be diluted, which simplifies the whole process even more.

The majority of laboratory errors occur in the preanalytical phase, with analytical interference and analyte stability being their main causes. In a clinical laboratory, it is essential to take into account these error sources to ensure reliable results, as we guarantee with our method. Importantly, prior reports about analogous methods lacked a well-detailed description of interfering compounds (Figure 1) and a short stability study (Figure 2).

There may be some possible limitations in this study. First, one transition for both busulfan and IS was used, and ion ratios were not calculated to detect potential interferences. The second limitation concerns the carryover study. Higher concentrations that may be encountered in clinical samples drawn through the intravenous line were not included in this assay.

This study was supported by professionals from the Pediatric Onco-Hematology Unit, and this method is currently being implemented into our health-care practice. Our effort will allow future pharmacokinetic studies to be designed in a way that minimizes toxicity, increases the efficacy of treatments, and improves the outcomes of transplants performed in our Center.

## Supplementary Material

Supplementary Material
