# Supplementary material for: Rapid and accurate method for quantifying busulfan in plasma samples by isocratic liquid chromatography-tandem mass spectrometry (LC-MS/MS)
Source: Adv Lab Med. 2022 Jun 13;3(3):263–71. doi: 10.1515/almed-2022-0016 (PMC10197276; doi:10.1515/almed-2022-0016)
Supplement: Supplementary file 1 — Supplementary Material [file j_almed-2022-0016_suppl.zip › Supplemental Table 1.docx]

**Supplemental Table 1**. Validation results of accuracy (n=15), intra-day precision (n=15) and inter-day precision (n=15).

| Spiked samples (mg/L) | Intra-day CV^†^ (%) | Inter-day CV^†^ (%) | Overall Bias^‡^ (%) |
| --- | --- | --- | --- |
| LLOQ (0.03) | 6.38 | 6.00 | 2.7 |
| VERY LOW (0.09) | 5.61 | 7.20 | 9.6 |
| LOW (0.75) | 6.13 | 7.08 | 9.0 |
| MEDIUM (1.50) | 5.85 | 5.01 | 5.6 |
| HIGH (4.00) | 4.89 | 6.63 | 8.3 |

^†^CV = Coefficient of variation;

^‡^Bias calculated as 100*(Measured concentration - Nominal concentration)/Nominal concentration.
