# Supplementary figures and images for: Rapid and accurate method for quantifying busulfan in plasma samples by isocratic liquid chromatography-tandem mass spectrometry (LC-MS/MS)
Source: Adv Lab Med. 2022 Jun 13;3(3):263–71. doi: 10.1515/almed-2022-0016 (PMC10197276; doi:10.1515/almed-2022-0016)

## Slide 1
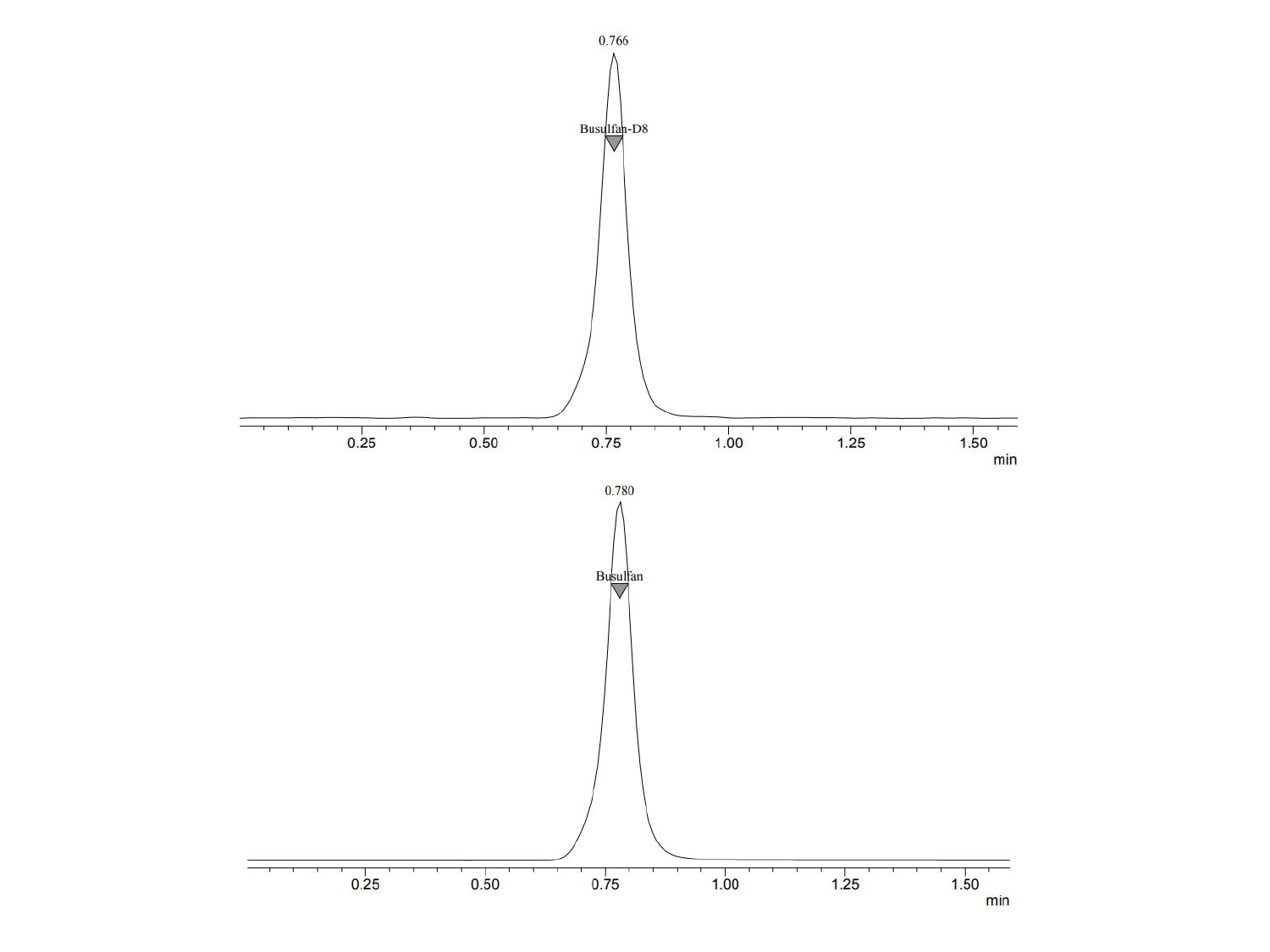

Supplement: Supplementary file 1 — Supplementary Material [file j_almed-2022-0016_suppl.zip › Supplemental Figure 1.pptx]

## Slide 1
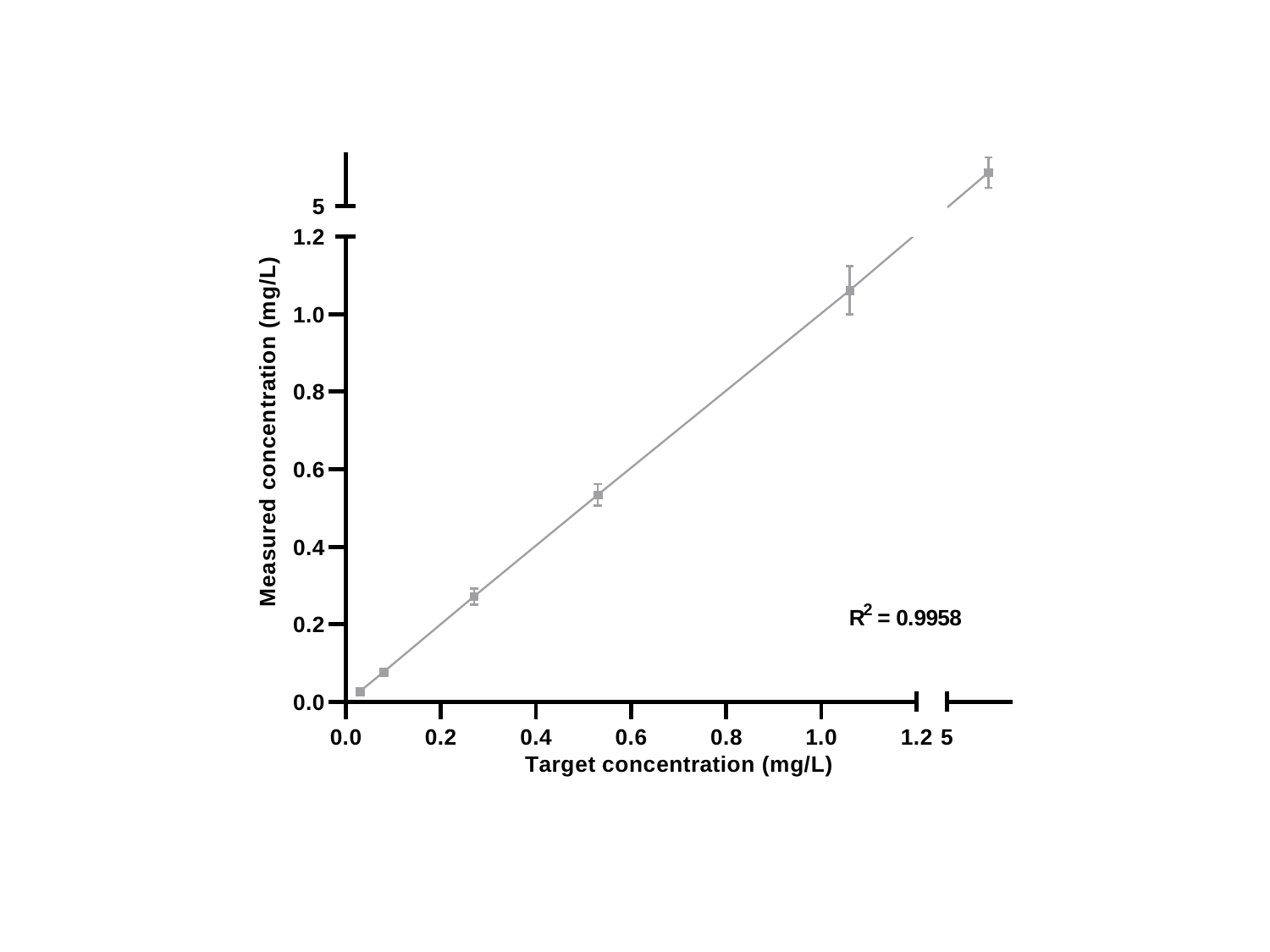

Supplement: Supplementary file 1 — Supplementary Material [file j_almed-2022-0016_suppl.zip › Supplemental Figure 2.pptx]
